# Serum Galectin-1 as a Diagnostic Biomarker in Endometriosis: A Prospective Longitudinal Study

**DOI:** 10.3390/ijms262110390

**Published:** 2025-10-25

**Authors:** Reka Brubel, Dora Bianka Balogh, Beata Polgar, Laszlo Szereday, Gernot Hudelist, Nandor Acs, Attila Bokor

**Affiliations:** 1 Department of Obstetrics and Gynecology, Semmelweis University, 1085 Budapest, Hungary; balogh.dora.bianka@semmelweis.hu (D.B.B.);; 2 Department of Medical Microbiology, Medical School, University of Pecs, 7626 Pecs, Hungary; 3 Department of Gynaecology, Centre for Endometriosis, Hospital St. John of God, 1020 Vienna, Austria; 4 Rudolfinerhaus Private Clinic and Campus, 1190 Vienna, Austria

**Keywords:** galectin, endometriosis, non-invasive diagnosis

## Abstract

Endometriosis is a chronic condition characterized by the presence of endometrial-like tissue outside the uterine cavity. It affects ~10% of reproductive-aged individuals and is associated with dysmenorrhea and infertility. Although imaging modalities have improved diagnosis, laparoscopy is required in many cases, contributing to 4–11 years of diagnostic delay. Non-invasive biomarkers could improve diagnosis and clinical decision-making, yet no candidate has achieved sufficient accuracy for routine use. Galectins, a family of β-galactoside-binding lectins involved in angiogenesis, immune regulation, and fibrosis, have emerged as promising biomarkers. In this study, we measured serum Galectin-1 (Gal-1) concentrations in 80 women with endometriosis and 15 controls using ELISA at four time points. Preoperative Gal-1 levels were significantly higher in endometriosis patients, particularly in Stage III–IV disease. ROC analysis yielded a modest diagnostic performance (AUC 0.692; *p* = 0.011) with high sensitivity (91.3%) and excellent negative predictive value (96.8%) but low specificity (46.7%) at a study-derived threshold (>14.06 ng/mL). Longitudinally, Gal-1 levels decreased immediately after surgery and rose above baseline by one year, while no significant correlations with preoperative pain severity were observed. These findings suggest that serum Gal-1 alone is insufficient as a diagnostic test but may be useful for multi-marker strategies to improve early diagnosis.

## 1. Introduction

Endometriosis is a chronic gynecological condition defined by endometrial-like tissue growth outside the uterine cavity. It affects approximately 10% of individuals of reproductive age, corresponding to 190 million people worldwide [1,2]. The disease manifests with a heterogeneous clinical spectrum, including dysmenorrhoea, chronic pelvic pain, dyspareunia, fatigue, heavy menstrual bleeding, and infertility [3,4,5]. Despite its high prevalence and substantial impact on quality of life, endometriosis remains underdiagnosed, with a reported diagnostic delay ranging from 4 to 11 years from symptom onset [6,7]. Multiple factors contribute to this delay, including the lack of reliable non-invasive biomarkers, the overlap of symptoms with other gynecological and gastrointestinal disorders—such as irritable bowel syndrome, interstitial cystitis, and primary dysmenorrhoea—and the reliance on surgical confirmation in many cases [8,9,10].

Achieving a timely and accurate diagnosis, therefore, remains challenging. Although advances in imaging techniques, particularly transvaginal ultrasound, have improved preoperative detection, these methods remain limited in sensitivity and specificity [6,11]. Therefore, there is an urgent need for novel, non-invasive biomarkers to improve diagnostic precision and enable earlier intervention.

Galectins, a family of β-galactoside-binding lectins, have emerged as significant contributors to endometriosis pathogenesis. These multifunctional proteins regulate key processes central to endometriosis pathophysiology, including immune cell survival, angiogenesis, cell adhesion, and tissue remodeling—mechanisms critical for the establishment and persistence of ectopic lesions. Among them, Galectin1 (Gal-1), Galectin-3 (Gal-3), and Galectin-9 (Gal-9) are expressed in the endometrium and decidua, and Gal 1–4, 8–9, 12–14, and -17 at the fetal–maternal interface [12,13].

Several studies have demonstrated that Gal-1 is increased at the cellular and tissue level in endometriosis. Immunohistochemistry and immunoblotting show overexpression of Gal-1 in ectopic lesions compared with paired eutopic endometrium from the same patients, and higher levels in the eutopic endometrium of women with endometriosis than in healthy controls, with stromal and endothelial cells as prominent sources [14]. Genetic deletion of the *Lgals1* gene or neutralization with an anti-Gal-1 monoclonal antibody reduced lesion size and vascularized area within the peritoneal cavity, with effects occurring independently of vascular endothelial growth factor (VEGF) signaling, underscoring Gal-1 as a driver of lesion angiogenesis and maintenance [15]. This aligns with broader evidence that angiogenesis is required for ectopic implant survival and expansion. Taken together, these studies provide lesion-level and cell-type evidence that Gal-1 is enriched in endometriosis and actively promotes angiogenesis and immune deviation, biological axes that underlie lesion establishment. Beyond its vascular functions, Gal-1 mediates peripheral immune tolerance and promotes fibroblast-to-myofibroblast transition, implicating it in tissue remodeling observed in endometriotic lesions [16,17].

Building on this biological rationale, our group has previously explored the potential diagnostic utility of other galectins in endometriosis. In an earlier study, we demonstrated that Gal-9 mRNA is expressed in ectopic implants and peritoneal cells, and that serum Gal-9 concentrations are significantly elevated in patients with endometriosis, with excellent diagnostic performance (AUC of 0.973, sensitivity of 94%, and specificity of 93.8%) [18]. More recently, we investigated serum Gal-3 in endometriosis and found increasing levels during longitudinal follow-up, suggesting its potential role in diagnosis and disease monitoring [19].

Despite these advances, the clinical utility of Gal-1 as a circulating biomarker for endometriosis remains unexplored. No study has systematically evaluated serum Gal-1 concentrations in a prospective, surgery-verified cohort or assessed its diagnostic accuracy. Therefore, this study aimed to quantify preoperative and longitudinal changes in serum Gal-1 levels in patients with histologically confirmed endometriosis compared with healthy controls, evaluate its diagnostic performance, and assess associations with preoperative symptom burden.

## 2. Results

### 2.1. Serum Gal-1 Levels in Patients with Endometriosis and Healthy Controls

**Figure 1:**
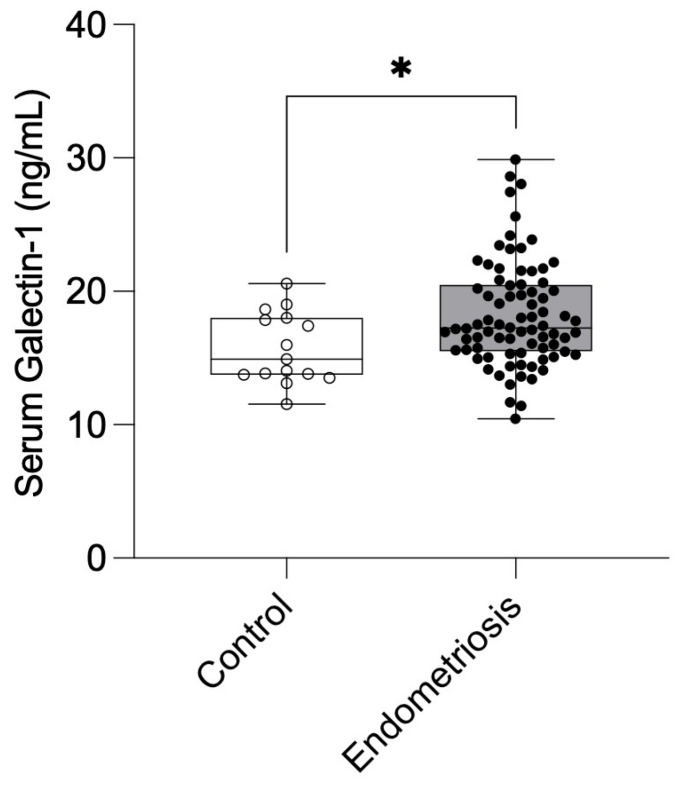
Preoperative serum Galectin-1 levels in healthy controls and endometriosis patients. Galectin-1 concentrations were measured in healthy individuals (n = 15) and patients with endometriosis (Stage I–IV; n = 80) before surgery. Data are presented as mean ± SD. Statistical comparisons were performed using the Mann–Whitney U test. * *p* < 0.05 vs. control.

Serum Gal-1 concentrations were significantly higher in patients with endometriosis compared with healthy controls. The mean preoperative Gal-1 concentration in the endometriosis cohort (n = 80) was 18.22 ± 3.94 ng/mL (95% CI: 17.35–19.10), whereas healthy control samples (n = 15) showed a mean level of 15.74 ± 2.66 ng/mL (95% CI: 14.26–17.21; *p* = 0.030) (Figure 1).

### 2.2. Serum Gal-1 Levels Based on the Severity of Endometriosis

**Figure 2:**
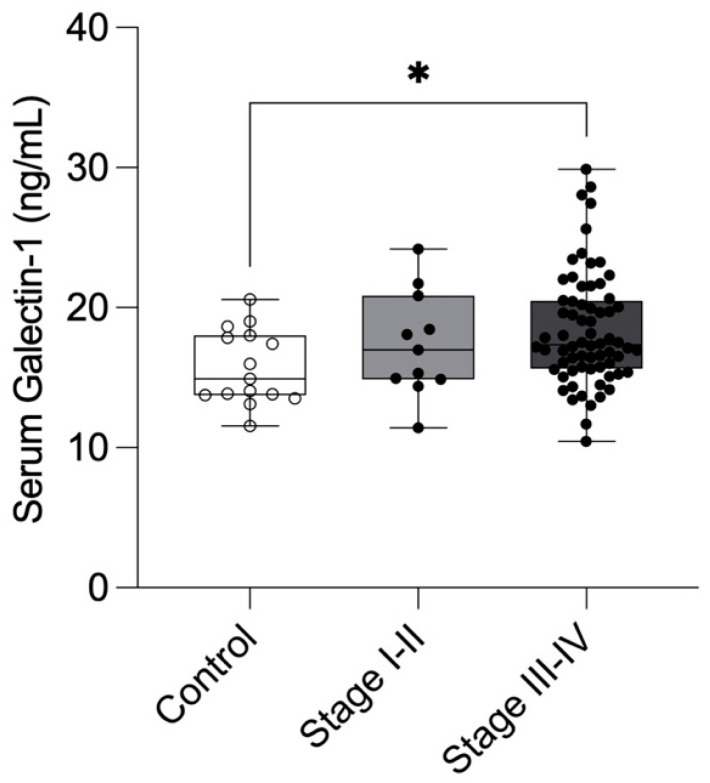
Preoperative serum Galectin-1 levels in healthy controls and patients with minimal-mild (Stage I–II) and moderate-severe (Stage III–IV) endometriosis. Galectin-1 concentrations were measured in healthy individuals (n = 15), patients with minimal-mild endometriosis (Stage I–II; n = 11), and patients with moderate-severe endometriosis (Stage III–V; n = 69) before surgery. Data are presented as mean ± SD. Statistical analysis was performed using the Kruskal–Wallis test with Dunn’s multiple comparisons. * *p* < 0.05 vs. control.

Our results demonstrated significantly elevated serum Gal-1 levels in patients with moderate-severe endometriosis (Stage III–IV; 18.4 ± 3.98 ng/mL, 95% CI: 17.4–19.3) compared with healthy controls (15.7 ± 2.66 ng/mL, 95% CI: 14.3–17.2, n = 15; *p* = 0.0299). Patients with minimal-mild endometriosis (Stage I–II; 17.38 ± 3.74 ng/mL, 95% CI: 14.87–19.89) also exhibited higher Gal-1 concentrations than controls, though this difference was not statistically significant (Figure 2).

### 2.3. Longitudinal Changes in Serum Gal 1 Levels During Patient Follow-Up

**Figure 3:**
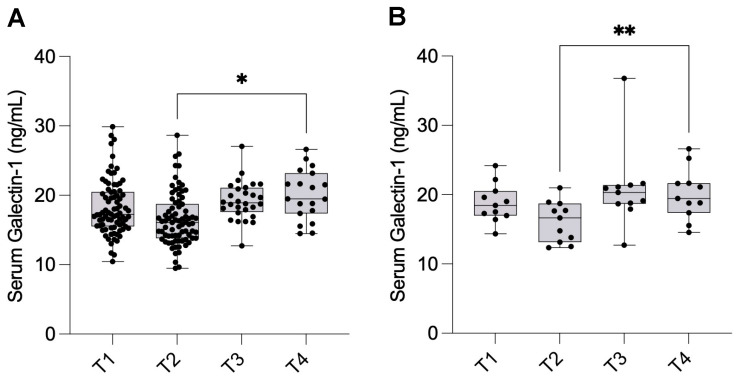
Longitudinal changes in serum Galectin-1 levels in endometriosis patients. (**A**) Serum Galectin-1 levels were measured in patients with endometriosis before surgery (T1; n = 80), after surgery (T2; n = 78), after one month (T3; n = 28), and after one year (T4; n = 19). Final sample sizes reflect data after outlier exclusion. Data are presented as mean ± SD. Statistical analysis was performed using mixed-effects analysis with Geisser–Greenhouse correction and Holm–Sídák multiple comparisons (using individual variances for each comparison). (**B**) A Friedman test was conducted as a sensitivity analysis on the subset of patients with complete data at all time points (T1-T4; n = 11). * *p* < 0.05, ** *p* < 0.01.

The mean serum Gal-1 levels were 18.2 ± 3.94 ng/mL (95% CI: 17.4–19.1) at T1, which slightly decreased to 16.6 ± 3.79 ng/mL (95% CI: 15.7–17.4) at T2. At one month postoperatively (T3), the mean Gal-1 level increased to 19.2 ± 2.74 ng/mL (95% CI: 18.2–20.3). A further increase was observed one year after surgery (T4), reaching 20.1 ± 3.60 ng/mL (95% CI: 18.3–21.8). Serum Gal-1 levels were significantly elevated at T4 compared to T2 (*p* = 0.0231) (Figure 3A).

Given patient attrition during follow-up, a sensitivity analysis was performed to confirm the robustness of our findings. A complete case analysis of the subset of 11 patients who provided data at all four time points revealed similar trends in Gal-1 levels, with mean values of 18.8 ± 2.77 ng/mL (95% CI: 16.9–20.6) at T1, decreasing to 16.1 ± 2.94 ng/mL (95% CI: 14.1–18.1) at T2, followed by an increase at T3 (20.9 ± 5.85 ng/mL, 95% CI: 16.9–24.8), and a stable elevation at T4 (20.1 ± 3.70 ng/mL, 95% CI: 17.6–22.6). The Friedman test confirmed statistically significant changes at T4 compared to T2 (*p* = 0.0089), reinforcing the validity of our primary findings (Figure 3B).

### 2.4. Serum Gal-1 Levels During Follow-Up According to Disease Severity

When analyzing serum Gal-1 levels separately according to disease severity, similar trends were observed in both groups, although the differences did not reach statistical significance.

**Figure 4:**
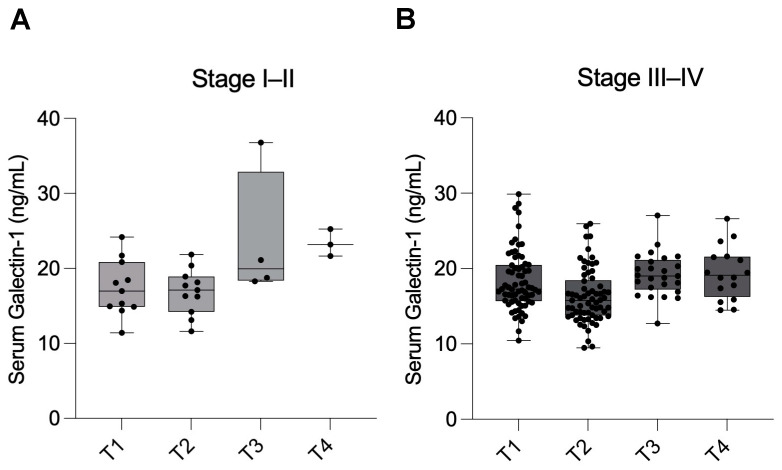
Longitudinal changes in serum Galectin-1 levels in patients with minimal-mild and moderate-severe endometriosis. Serum Galectin-1 levels were measured in patients with endometriosis at four time points: before surgery (T1), 1 day after surgery (T2), 1 month after surgery (T3), and 1 year after surgery (T4). (**A**) Serum Galectin-1 levels in patients with minimal-mild endometriosis (Stage I–II) at T1 (n = 11), T2 (n = 11), T3 (n = 4), and T4 (n = 3). (**B**) Serum Galectin-1 levels in patients with moderate-severe endometriosis (Stage III–IV) at T1 (n = 69), T2 (n = 66), T3 (n = 25), and T4 (n = 16). Final sample sizes reflect data after outlier exclusion. Data are presented as mean ± SD. Statistical analysis was performed using mixed-effects analysis with Geisser–Greenhouse correction and Holm–Sídák multiple comparisons (using individual variances for each comparison).

In patients with minimal-mild endometriosis (Stage I–II), Gal-1 levels were 17.4 ± 3.74 ng/mL (95% CI: 14.9–19.9) at T1, slightly decreasing to 16.9 ± 3.05 ng/mL (95% CI: 14.8–18.9) at T2. Levels showed an apparent increase at T3 (23.7 ± 8.79 ng/mL, 95% CI: 9.7–37.7) and remained high at T4 (23.4 ± 1.80 ng/mL, 95% CI: 18.9–27.8) (Figure 4A). Due to substantial attrition over the follow-up period, Stage I–II subgroup analyses should be interpreted cautiously and considered exploratory.

In patients with moderate-severe endometriosis (Stage III–IV), serum Gal-1 levels were 18.5 ± 3.99 ng/mL (95% CI: 17.5–19.4) at T1, decreasing to 16.4 ± 3.62 ng/mL (95% CI: 15.5–17.3) at T2. Levels increased again at T3 (19.2 ± 2.87 ng/mL, 95% CI: 18.0–20.4) and remained elevated at T4 (19.1 ± 3.41 ng/mL, 95% CI: 17.2–21.0) (Figure 4B).

The lack of significant differences within subgroups, particularly the Stage I–II group, may be attributed to the limited sample size during the follow-up period.

### 2.5. Diagnostic Performance of Serum Gal-1 Levels in Endometriosis

**Figure 5:**
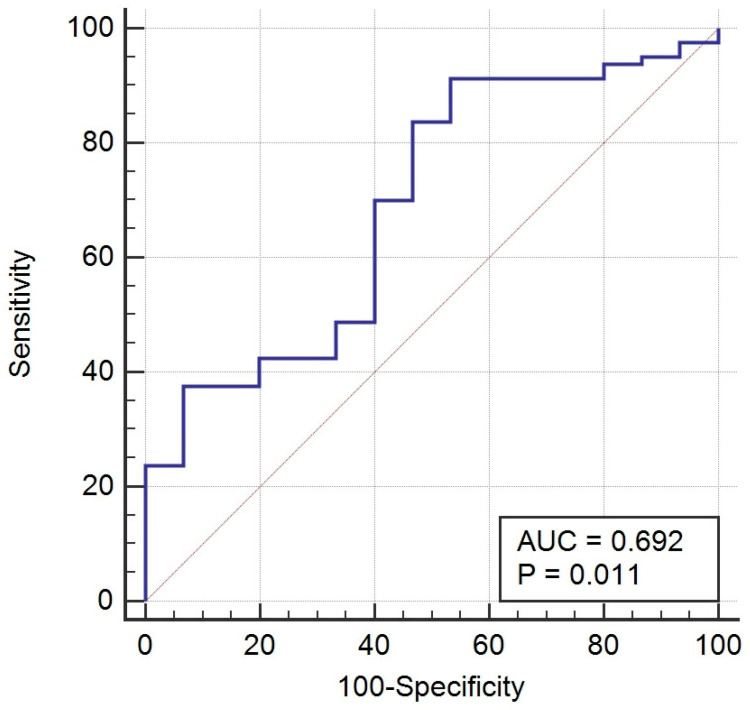
Receiver operating characteristic (ROC) curve analysis of serum Galectin-1 in endometriosis vs. healthy controls. The ROC curve illustrates the diagnostic performance of serum Galectin-1 levels in distinguishing patients with endometriosis (n = 80) from healthy controls (n = 15). The area under the curve (AUC) was 0.692 (*p* = 0.011).

To evaluate the potential of serum Gal-1 as a biomarker for diagnosing endometriosis, we performed a receiver operating characteristic (ROC) curve analysis comparing Gal-1 levels between patients with endometriosis and healthy controls. As shown in Figure 5, the area under the curve (AUC) was 0.692 (95% CI: 0.54–0.83; *p* = 0.011), indicating a modest ability of serum Gal-1 to distinguish between the two groups. At the optimal cut-off value (>14.06 ng/mL), serum Gal-1 demonstrated a high sensitivity (91.3%) but a relatively low specificity (46.7%). At this threshold, the positive predictive value (PPV) was 23.19%, while the negative predictive value (NPV) was notably high at 96.8%. However, the overall diagnostic accuracy was modest at 53.4%.

### 2.6. Serum Gal-1 Levels Are Not Associated with Endometriosis-Related Pain Severity

**Figure 6:**
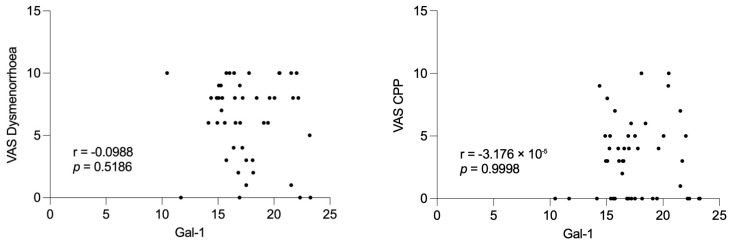

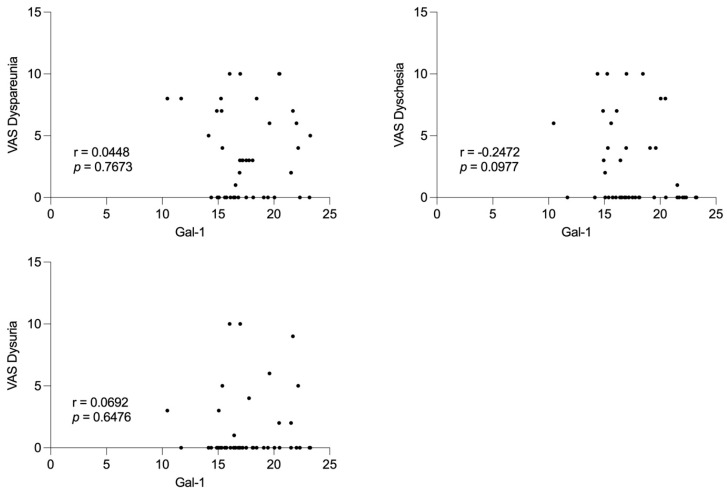
Correlation between preoperative serum Galectin-1 (Gal-1) levels and endometriosis-associated pain scores. Scatter plots show the relationship between serum Gal-1 concentrations and visual analogue scale (VAS) scores for dysmenorrhoea, chronic pelvic pain (CPP), dyspareunia, dyschesia, and dysuria in patients with endometriosis (n = 46). Spearman’s rank correlation coefficients (r) and corresponding *p*-values are displayed for each symptom.

We evaluated the relationship between preoperative serum Gal-1 concentrations and patient-reported pain severity using a visual analog scale (VAS). As shown in Figure 6, there were no statistically significant correlations between Gal-1 levels and any of the assessed endometriosis-related pain domains, including dysmenorrhoea (r = −0.099, *p* = 0.519), chronic pelvic pain (CPP; r ≈ 0.000, *p* = 0.999), dyspareunia (r = 0.045, *p* = 0.767), dyschesia (r = −0.247, *p* = 0.098), and dysuria (r = 0.069, *p* = 0.648). These findings indicate that serum Gal-1 levels do not reflect patient-reported pain severity in endometriosis.

## 3. Discussion

Endometriosis diagnosis remains challenging. Despite improvements in high-resolution transvaginal ultrasound and MRI, definitive confirmation often still relies on laparoscopy, particularly for minimal and superficial disease [6]. As a result, many women experience a substantial diagnostic delay, ranging from 4 to 11 years in adults, and up to 7 years in adolescents [20,21]. Such a long delay often pushes clinical management into empirical or late-stage interventions, limiting opportunities for timely surgery or fertility preservation. This problem is particularly critical in adolescents, where atypical or non-cyclic pain presentations frequently contribute to diagnostic uncertainty. Prolonged exposure to pelvic pain during adolescence can sensitize the nervous system and increase the risk of developing chronic pelvic pain and other central sensitization syndromes later in life [21,22]. These challenges underscore the need for non-invasive, cost-effective, and reproducible biomarkers that can reliably differentiate disease stages and monitor progression [6,23].

Despite extensive research, no single serum biomarker has achieved sufficient diagnostic accuracy to replace surgical confirmation. Nisenblat et al. concluded that markers, including cancer antigen 125 (CA-125), inflammatory cytokines, growth factors, and angiogenic mediators, have limited sensitivity and specificity, especially in minimal–mild disease [9]. Similarly, another review emphasized the heterogeneity across study designs, assay methodologies, and patient phenotypes, limiting the translation of findings into clinical practice [24]. These limitations underscore the urgent need for novel biomarker candidates and multi-marker strategies that capture distinct biological pathways underlying endometriosis pathogenesis.

Our prospective study found that preoperative serum Gal-1 concentrations were significantly elevated in women with endometriosis compared to healthy controls, particularly in those with Stage III–IV disease. This suggests that serum Gal-1 levels may reflect disease severity in endometriosis. The ROC analysis indicated a modest diagnostic performance (AUC = 0.692, *p* = 0.011), characterized by high sensitivity (91.3%), but limited specificity (46.7%). Accordingly, NPV was very high (96.8%), while PPV remained modest (23.2%). Although Gal-1 demonstrated a statistically significant difference and a modest discriminative performance in endometriosis, its current diagnostic performance does not support clinical application as a single biomarker. Nonetheless, the observed elevation in Gal-1 levels among affected patients may still provide insight into disease pathophysiology and could contribute to improved diagnostic accuracy when combined with other promising biomarkers or imaging findings.

The relatively low specificity of Gal-1 observed in our study likely reflects both biological and methodological factors. Biologically, Gal-1 is a multifunctional molecule involved in immune regulation, angiogenesis, and tissue remodeling; its upregulation is not specific to endometriosis but also occurs in various inflammatory and neoplastic conditions. Consequently, circulating levels may be elevated in individuals without endometriosis but with other sources of inflammation. Methodologically, the small number of healthy controls and potential variability related to menstrual phase or hormonal status could have contributed to the observed overlap between groups.

Longitudinal analyses revealed a transient postoperative decrease in Gal-1 levels one day after surgery, followed by a rise above baseline levels at one year. It is well established that complete surgical eradication of all endometriotic tissue is rarely achievable. Even with meticulous excision, microscopic or deeply infiltrating foci often remain undetected intraoperatively, particularly in anatomically complex regions such as the rectovaginal septum, bowel, or pelvic sidewall. Endometriosis recurrence can result from de novo lesion formation and the in situ growth of residual microscopic implants that were not visible or accessible during surgery [25]. Similarly, a comprehensive review emphasized that recurrent disease frequently reflects the persistence and reactivation of these residual endometriotic cells or micro-lesions, rather than disease reappearance [26]. In this context, the postoperative rise in Gal-1 levels observed one year after surgery may partially reflect ongoing biological activity from residual endometriotic tissue or associated low-grade inflammation and tissue remodeling. This interpretation aligns with the known functions of Gal-1 in angiogenesis, immune modulation, and fibrosis, supporting its detectability as a systemic indicator of persistent or residual disease activity rather than a definitive marker of recurrence.

The lack of significant differences within the subgroups according to disease severity, particularly the Stage I–II group, may be attributed to the limited sample size during the follow-up period. Notably, Gal-1 concentrations were not associated with preoperative pain severity, suggesting that Gal-1 may reflect disease activity rather than subjective symptom perception.

Our findings are supported by mechanistic data implicating Gal-1 in endometriosis pathophysiology [13]. Ectopic stromal and endothelial cells overexpress Gal-1, which is further induced by neuropeptides such as corticotropin-releasing hormone and urocortin, linking the neuroendocrine system to local immune modulation [14]. Experimental studies confirm that Gal-1 deficiency or neutralization dampens lesion angiogenesis and growth, independently of VEGF, highlighting its functional centrality in lesion persistence [15]. The observed perioperative fluctuations in serum Gal-1 may reflect the biological response to surgical lesion removal and postoperative remodeling, supporting its systemic detectability as a marker of disease activity rather than a specific indicator of recurrence.

The first clinical data for the systemic involvement of galectins in endometriosis came from our case–control study, which demonstrated that serum Gal-9 levels are significantly elevated in patients with endometriosis compared to healthy controls [18]. These findings were subsequently confirmed by an independent study using laparoscopy-confirmed diagnoses [27]. Building on these insights, we expanded our investigations to Gal-3 in a longitudinal study, showing that serum Gal-3 levels are elevated in endometriosis and increase progressively during postoperative follow-up, suggesting a potential role not only as a diagnostic marker but also for monitoring disease dynamics [19]. Collectively, these studies highlight the promise of galectins as non-invasive biomarkers in endometriosis and provide the rationale for investigating Gal-1 in this prospective cohort.

Given the limited performance of single biomarkers, integrating markers that represent complementary biological pathways may enhance diagnostic accuracy. For instance, panels combining CA-125, VEGF, Annexin V, and glycodelin/sICAM-1 have demonstrated a sensitivity of 74–94% and a specificity of 55–75% outperforming individual markers [28]. Similarly, IL-6, prolactin, and CA-125 combinations have shown improved advanced disease detection [29]. However, none of these biomarker panels has yet been translated into routine clinical practice.

While our findings support the role of Gal-1 as a potential disease-related biomarker, its standalone diagnostic performance is insufficient for clinical application. Since Gal-1 mediates angiogenesis and immune tolerance biology, its integration with Gal-3 (fibrosis and extracellular matrix remodeling), Gal-9 (immune checkpoint signaling), and established markers such as CA-125 and inflammatory cytokines can improve diagnostic sensitivity. Embedding such panels into predictive algorithms, alongside clinical and imaging data, might enable personalized triage to laparoscopy and facilitate earlier treatment planning. Future studies should investigate whether multi-marker panels incorporating Gal-1 could improve diagnostic performance. To ensure reproducibility, such work will require validation using standardized assays, prespecified cut-offs, and calibration across clinical centers.

The strengths of this study include its prospective design with surgery- and histology-confirmed diagnoses, the use of a standardized ELISA protocol with clearly defined assay performance characteristics, and the inclusion of longitudinal sampling across four perioperative time points, which allowed us to assess dynamic changes in serum Gal-1 levels over time. Furthermore, we applied comprehensive diagnostic performance analyses, including ROC-based thresholds, sensitivity, specificity, and predictive values, which provide a transparent clinical framework for interpretation. Nevertheless, several limitations should be acknowledged. The control group was relatively small and lacked complete demographic data due to anonymized recruitment through the national blood bank, which limits generalizability. Attrition at later follow-up points reduced statistical power for subgroup analyses, particularly in Stage I–II disease. The study cohort was imbalanced toward Stage III–IV disease, which may have influenced overall diagnostic performance. The low specificity observed in ROC analysis indicates that serum Gal-1 alone is unsuitable for clinical application and should be viewed as a complementary rather than a standalone biomarker. Finally, our findings are based on a single-center design without external validation. The findings should be considered exploratory. Future studies should include larger, multicenter cohorts with symptomatic and healthy controls to enhance statistical robustness and external validity.

## 4. Materials and Methods

### 4.1. Ethical Approval and Patient Enrollment

**Figure 7:**
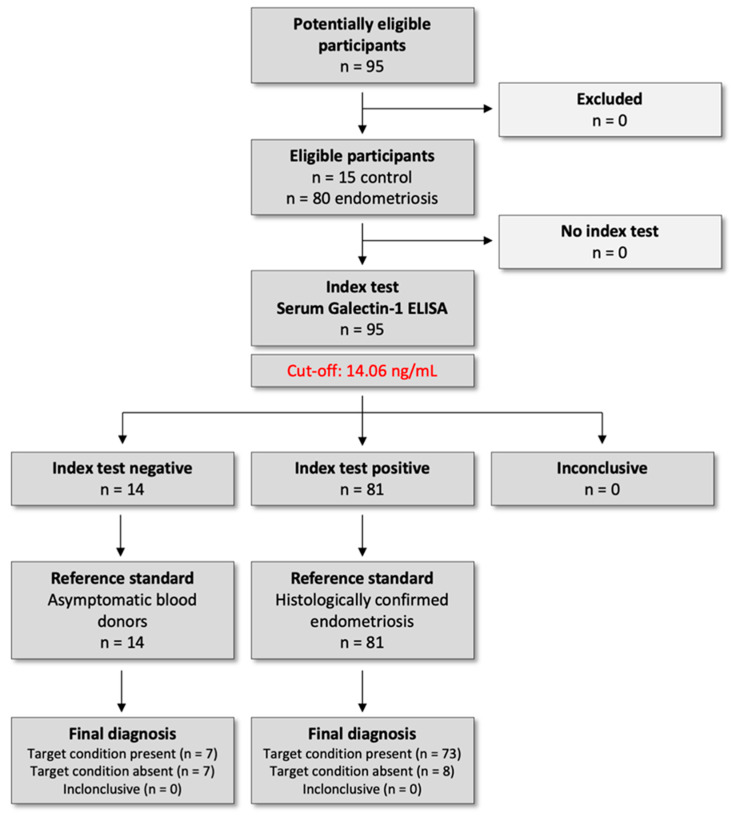
STARD 2015 study flow diagram for diagnostic accuracy of serum Galectin-1 in endometriosis.

The study protocol was approved by the Institutional Ethical and Review Board of Semmelweis University, Budapest (registration no. 143/2008, 15 October 2008), and was conducted per the Declaration of Helsinki. All participants provided written informed consent before inclusion in this prospective case–control study. This study was conducted as part of a registered clinical trial in the ClinicalTrials.gov registry (U.S. National Library of Medicine), under the identifier NCT04401592 on 1 May 2020 (https://clinicaltrials.gov/study/NCT04401592). We followed the Standards for Reporting Diagnostic Accuracy Studies (STARD) 2015 guidelines to ensure transparent reporting of study design, conduct, and diagnostic performance analyses (Figure 7).

Participants were consecutively recruited between 1 September 2021 and 1 September 2023. A total of 80 reproductive-aged women undergoing laparoscopy for pelvic pain and/or infertility were enrolled. Age-matched healthy female blood donors (n = 15) were recruited anonymously through the National Blood Bank Regional Centre in Budapest and served as controls. In addition to fulfilling the standard eligibility criteria for blood donation, all participants completed a self-reported health questionnaire confirming the absence of any known gynecological, infectious, inflammatory, or systemic conditions at the time of donation. These procedures ensured the inclusion of a generally healthy donor population. However, as the samples were collected anonymously, detailed demographic and clinical background data were unavailable.

### 4.2. Inclusion and Exclusion Criteria

Women aged 15–40 years who underwent laparoscopy for suspected endometriosis at the Department of Obstetrics and Gynecology, Semmelweis University, were eligible for inclusion.

To ensure unbiased biomarker assessment, only patients who were not receiving any form of hormonal therapy prior to surgery were included. This restriction minimized potential hormonal effects on circulating Gal-1 levels.

Exclusion criteria comprised ongoing pregnancy, lactation, gynecologic malignancies, acute pelvic infections, or any known autoimmune, inflammatory, or metabolic disorders that could confound serum biomarker interpretation. Patients with incomplete perioperative data or insufficient serum sample volume were also excluded from the final analysis.

### 4.3. Clinical Classification of Endometriosis

Endometriosis was diagnosed and classified with laparoscopy performed at the Department of Obstetrics and Gynecology, Semmelweis University, with histological confirmation of endometriotic lesions obtained from surgical biopsies by the Department of Pathology and Experimental Cancer Research. All laparoscopic procedures were performed by experienced gynecologic surgeons specialized in minimally invasive endometriosis surgery, ensuring consistent diagnostic and sampling standards.

Following histopathological confirmation, patients were classified according to the Revised American Society for Reproductive Medicine (rASRM, 1997) staging system, which evaluates lesion size, depth of invasion, ovarian involvement, and the extent of adhesions. Based on this scoring, participants were categorized into minimal–mild (Stage I–II; n = 11) and moderate–severe (Stage III–IV; n = 69) disease groups.

Detailed intraoperative findings were recorded in the clinical database to ensure accurate staging, including lesion location, adhesion severity, and ovarian endometrioma presence. This stratification was subsequently used to compare serum Gal-1 levels between disease severity groups.

### 4.4. Pain Assessment

Preoperatively, pain intensity was evaluated using a visual analog scale (VAS) to capture the subjective severity of endometriosis-associated pain symptoms. Patients were asked to rate the intensity of five common pain domains—dysmenorrhoea, chronic pelvic pain, dyspareunia, dyschezia, and dysuria—on a continuous scale from 0 (no pain) to 10 (worst imaginable pain). The VAS was administered under standardized conditions during preoperative consultation to ensure consistency in reporting. Each symptom score was recorded separately. Higher values indicated greater pain severity.

### 4.5. Sample Collection

Serum samples for Gal-1 analysis were collected at four predefined time points to enable longitudinal analysis of serum Gal-1 levels over the perioperative and postoperative period: one day before surgery (T1, n = 80), one day after surgery (T2, n = 79), one month after surgery (T3, n = 28), and one year after surgery (T4, n = 19). The same patient cohort was followed prospectively throughout the study, and repeated samples were collected from each individual at all four time points. However, due to patient attrition during the follow-up period, the number of available samples decreased over time. The longitudinal design allowed for intra-individual comparison of serum Gal-1 concentrations, capturing short-term postoperative changes as well as long-term biological dynamics up to one year after surgical treatment.

### 4.6. Serum Preparation

Blood samples were allowed to clot at room temperature, ensuring complete coagulation before processing. Then, the tubes were centrifuged at 3000 rpm for 10 min to separate serum from cellular components. The supernatant was carefully aspirated to avoid contamination with the buffy coat and aliquoted into microtubes.

To preserve sample integrity, all serum aliquots were immediately stored at −80 °C until biochemical analysis. This protocol minimized potential degradation of Gal-1 and prevented protein denaturation due to temperature fluctuation. Each sample was subjected to a single controlled freeze–thaw cycle before measurement. Pre-analytical sample handling, storage, and thawing were performed according to standardized laboratory quality control procedures to ensure data reliability and reproducibility.

### 4.7. Enzyme-Linked Immunosorbent Assay (ELISA) Measurements of Gal-1

Serum Gal-1 levels were determined using a human Gal-1-specific ELISA kit (Quantikine Human Gal-1 ELISA kit, R&D Systems, Minneapolis, MN, USA; Cat. no: DGAL10), following the manufacturer’s protocol. Optical density (OD) measurements were performed at 450 nm with a wavelength correction set at 540 nm using a SPECTROStar Nano microplate reader (BMG Labtech, Ortenberg, Germany). Gal-1 concentrations were calculated using a 4-parameter logistic regression fitted by MARS Data Analysis Software (version 3.32; BMG Labtech). Results are reported as ng/mL. The assay characteristics included a minimum detectable dose (MDD) of 0.022 ng/mL, intra-assay coefficient of variation (CV%) between 5.7 and 8.8%, inter-assay CV% between 7.5 and 9.5%, and a standard curve range of 0.313–20 ng/mL.

To ensure reliability and minimize inter- and intra-assay variability, all samples were analyzed in duplicate under identical experimental conditions. Calibrators containing human recombinant Gal-1 provided by the manufacturer were included in each plate. Potential sources of pre-analytical bias, such as matrix effects and potential interferences resulting from sample hemolysis, lipemia, freeze–thaw cycles, and prolonged storage, were strictly controlled. These measures strengthen the robustness of our findings and support the suitability of the Gal-1 ELISA for use in endometriosis research.

### 4.8. Statistical Analysis

Data are presented as mean ± standard deviation (SD). Statistical analyses were performed using GraphPad Prism (version 10.1.0; GraphPad Software, San Diego, CA, USA). ROC curve analysis was conducted with MedCalc (version 16.8; MedCalc Software Ltd., Ostend, Belgium). Descriptive statistics were computed for all variables, including mean, standard deviation, and confidence intervals. Outliers were identified and excluded using the robust regression and outlier removal (ROUT) method.

Between-group comparisons were performed using appropriate statistical tests based on data distribution and group numbers, including the unpaired *t*-test, Mann–Whitney U test, Kruskal–Wallis test with Dunn’s post hoc analysis, and mixed-effects analysis with Holm–Sídák multiple comparison tests. For repeated measures analyses, the Geisser–Greenhouse correction was applied when necessary to account for violations of sphericity assumptions. A Friedman test was conducted as a sensitivity analysis on the subset of patients with complete data at all time points.

The ROC curve analysis evaluated the diagnostic performance of Gal-1 for distinguishing endometriosis from healthy controls. Gal-1 levels from confirmed endometriosis cases were the positive group, while healthy controls provided the negative reference. The optimal cut-off was determined using Youden’s index.

Spearman’s rank correlation was used to investigate potential associations between serum Gal 1 levels and preoperative pain scores. Correlation coefficients and corresponding *p*-values were calculated to assess the strength and statistical significance of these relationships. The *p* < 0.05 was considered statistically significant.

## 5. Conclusions

This prospective study provides the first comprehensive evaluation of serum Gal-1 in a surgery-confirmed cohort of women with endometriosis. We demonstrate that Gal-1 levels are significantly elevated in affected patients compared to healthy controls and exhibit perioperative fluctuations that likely reflect lesion burden and residual biological activity. Although Gal-1 demonstrated high sensitivity and an excellent negative predictive value, its limited specificity and modest diagnostic accuracy indicate that it cannot serve as a standalone diagnostic marker. Instead, these findings highlight Gal-1’s potential role as a complementary component within a multi-marker framework, particularly for rule-out or triage applications. Future studies with larger, externally validated cohorts should explore Gal-1 in combination with other galectins, CA-125, and cytokines, as well as integrate imaging data to enhance diagnostic precision and support earlier management of endometriosis.

## Data Availability

The datasets used and analyzed during the current study are available from the corresponding author on reasonable request. The data are not publicly available due to privacy or ethical restrictions in DAS.

## Abbreviations

The following abbreviations are used in this manuscript:

AUC	Area Under the Curve
ASRM	American Society for Reproductive Medicine
CA-125	Cancer Antigen 125
CI	Confidence Interval
CPP	Chronic Pelvic Pain
CV	Coefficient of Variation
ELISA	Enzyme-Linked Immunosorbent Assay
Gal-1	Galectin-1
Gal-3	Galectin-3
Gal-9	Galectin-9
IL-6	Interleukin-6
MARS	Microplate Analysis and Regression Software
MDD	Minimum Detectable Dose
mRNA	Messenger Ribonucleic Acid
NPV	Negative Predictive Value
OD	Optical Density
PPV	Positive Predictive Value
ROC	Receiver Operating Characteristic
ROUT	Robust Regression and Outlier Removal
sICAM	Soluble Intercellular Adhesion Molecule-1
SD	Standard Deviation
VAS	Visual Analog Scale
VEGF	Vascular Endothelial Growth Factor

## References

[1] Zondervan K.T., Becker C.M., Missmer S.A. (2020). Endometriosis. N. Engl. J. Med..

[2] Saunders P.T.K., Horne A.W. (2021). Endometriosis: Etiology, pathobiology, and therapeutic prospects. Cell.

[3] Mathias S.D., Kuppermann M., Liberman R.F., Lipschutz R.C., Steege J.F. (1996). Chronic pelvic pain: Prevalence, health-related quality of life, and economic correlates. Obstet. Gynecol..

[4] Facchin F., Barbara G., Saita E., Mosconi P., Roberto A., Fedele L., Vercellini P. (2015). Impact of endometriosis on quality of life and mental health: Pelvic pain makes the difference. J. Psychosom. Obstet. Gynaecol..

[5] Meuleman C., Vandenabeele B., Fieuws S., Spiessens C., Timmerman D., D’Hooghe T. (2009). High prevalence of endometriosis in infertile women with normal ovulation and normospermic partners. Fertil. Steril..

[6] Becker C.M., Bokor A., Heikinheimo O., Horne A., Jansen F., Kiesel L., King K., Kvaskoff M., Nap A., Petersen K. (2022). ESHRE guideline: Endometriosis. Hum. Reprod. Open.

[7] Greene R., Stratton P., Cleary S.D., Ballweg M.L., Sinaii N. (2009). Diagnostic experience among 4,334 women reporting surgically diagnosed endometriosis. Fertil. Steril..

[8] Gupta D., Hull M.L., Fraser I., Miller L., Bossuyt P.M., Johnson N., Nisenblat V. (2016). Endometrial biomarkers for the non-invasive diagnosis of endometriosis. Cochrane Database Syst. Rev..

[9] Nisenblat V., Bossuyt P.M., Shaikh R., Farquhar C., Jordan V., Scheffers C.S., Mol B.W., Johnson N., Hull M.L. (2016). Blood biomarkers for the non-invasive diagnosis of endometriosis. Cochrane Database Syst. Rev..

[10] Nisenblat V., Prentice L., Bossuyt P.M., Farquhar C., Hull M.L., Johnson N. (2016). Combination of the non-invasive tests for the diagnosis of endometriosis. Cochrane Database Syst. Rev..

[11] Condous G., Gerges B., Thomassin-Naggara I., Becker C., Tomassetti C., Krentel H., van Herendael B.J., Malzoni M., Abrao M.S., Saridogan E. (2024). Non-invasive imaging techniques for diagnosis of pelvic deep endometriosis and endometriosis classification systems: An International Consensus Statement. Ultrasound Obstet. Gynecol..

[12] Jeschke U., Hutter S., Heublein S., Vrekoussis T., Andergassen U., Unverdorben L., Papadakis G., Makrigiannakis A. (2013). Expression and function of galectins in the endometrium and at the human feto-maternal interface. Placenta.

[13] Hisrich B.V., Young R.B., Sansone A.M., Bowens Z., Green L.J., Lessey B.A., Blenda A.V. (2020). Role of Human Galectins in Inflammation and Cancers Associated with Endometriosis. Biomolecules.

[14] Vergetaki A., Jeschke U., Vrekoussis T., Taliouri E., Sabatini L., Papakonstanti E.A., Makrigiannakis A. (2014). Galectin-1 overexpression in endometriosis and its regulation by neuropeptides (CRH, UCN) indicating its important role in reproduction and inflammation. PLoS ONE.

[15] Baston J.I., Baranao R.I., Ricci A.G., Bilotas M.A., Olivares C.N., Singla J.J., Gonzalez A.M., Stupirski J.C., Croci D.O., Rabinovich G.A. (2014). Targeting galectin-1-induced angiogenesis mitigates the severity of endometriosis. J. Pathol..

[16] Cedeno-Laurent F., Dimitroff C.J. (2012). Galectin-1 research in T cell immunity: Past, present and future. Clin. Immunol..

[17] Sundblad V., Morosi L.G., Geffner J.R., Rabinovich G.A. (2017). Galectin-1: A Jack-of-All-Trades in the Resolution of Acute and Chronic Inflammation. J. Immunol..

[18] Brubel R., Bokor A., Pohl A., Schilli G.K., Szereday L., Bacher-Szamuel R., Rigo J., Polgar B. (2017). Serum galectin-9 as a noninvasive biomarker for the detection of endometriosis and pelvic pain or infertility-related gynecologic disorders. Fertil. Steril..

[19] Brubel R., Polgar B., Szereday L., Balogh D.B., Toth T., Mate S., Csibi N., Dobo N., Hudelist G., Acs N. (2025). Alteration of Serum Gal-3 Levels in Endometrium-Related Reproductive Disorders. Int. J. Mol. Sci..

[20] Simoens S., Dunselman G., Dirksen C., Hummelshoj L., Bokor A., Brandes I., Brodszky V., Canis M., Colombo G.L., DeLeire T. (2012). The burden of endometriosis: Costs and quality of life of women with endometriosis and treated in referral centres. Hum. Reprod..

[21] Sieberg C.B., Lunde C.E., Borsook D. (2020). Endometriosis and pain in the adolescent- striking early to limit suffering: A narrative review. Neurosci. Biobehav. Rev..

[22] Katz J., Seltzer Z. (2009). Transition from acute to chronic postsurgical pain: Risk factors and protective factors. Expert. Rev. Neurother..

[23] Encalada Soto D., Rassier S., Green I.C., Burnett T., Khan Z., Cope A. (2022). Endometriosis biomarkers of the disease: An update. Curr. Opin. Obstet. Gynecol..

[24] O D.F., Flores I., Waelkens E., D’Hooghe T. (2018). Noninvasive diagnosis of endometriosis: Review of current peripheral blood and endometrial biomarkers. Best. Pract. Res. Clin. Obstet. Gynaecol..

[25] D’Hooghe T.M., Denys B., Spiessens C., Meuleman C., Debrock S. (2006). Is the endometriosis recurrence rate increased after ovarian hyperstimulation?. Fertil. Steril..

[26] Ceccaroni M., Bounous V.E., Clarizia R., Mautone D., Mabrouk M. (2019). Recurrent endometriosis: A battle against an unknown enemy. Eur. J. Contracept. Reprod. Health Care.

[27] Jarollahi S., Chaichian S., Jarollahi A., Hajmohammadi R., Mashayekhi R., Shahmohammadi F., Eslamivaghar M., Ghasemi Z. (2022). The diagnostic accuracy of galectin-9 for diagnosis of endometriosis in comparison with laparoscopy. J. Reprod. Infertil..

[28] Vodolazkaia A., El-Aalamat Y., Popovic D., Mihalyi A., Bossuyt X., Kyama C.M., Fassbender A., Bokor A., Schols D., Huskens D. (2012). Evaluation of a panel of 28 biomarkers for the non-invasive diagnosis of endometriosis. Hum. Reprod..

[29] Kokot I., Piwowar A., Jedryka M., Solkiewicz K., Kratz E.M. (2021). Diagnostic Significance of Selected Serum Inflammatory Markers in Women with Advanced Endometriosis. Int. J. Mol. Sci..

